# Color Processing in Zebrafish Retina

**DOI:** 10.3389/fncel.2018.00327

**Published:** 2018-10-03

**Authors:** April Meier, Ralph Nelson, Victoria P. Connaughton

**Affiliations:** ^1^Zebrafish Ecotoxicology, Neuropharmacology, and Vision Lab, Department of Biology, and Center for Behavioral Neuroscience, American University, Washington, DC, United States; ^2^Neural Circuits Unit, National Institute of Neurological Disorders and Stroke (NINDS), NIH, Bethesda, MD, United States

**Keywords:** *Danio rerio*, color vision, red, green, blue, ultraviolet

## Abstract

Zebrafish (*Danio rerio*) is a model organism for vertebrate developmental processes and, through a variety of mutant and transgenic lines, various diseases and their complications. Some of these diseases relate to proper function of the visual system. In the US, the National Eye Institute indicates >140 million people over the age of 40 have some form of visual impairment. The causes of the impairments range from refractive error to cataract, diabetic retinopathy and glaucoma, plus heritable diseases such as retinitis pigmentosa and color vision deficits. Most impairments directly affect the retina, the nervous tissue at the back of the eye. Zebrafish with long or short-wavelength color blindness, altered retinal anatomy due to hyperglycemia, high intraocular pressure, and reduced pigment epithelium are all used, and directly applicable, to study how these symptoms affect visual function. However, many published reports describe only molecular/anatomical/structural changes or behavioral deficits. Recent work in zebrafish has documented physiological responses of the different cell types to colored (spectral) light stimuli, indicating a complex level of information processing and color vision in this species. The purpose of this review article is to consolidate published morphological and physiological data from different cells to describe how zebrafish retina is capable of complex visual processing. This information is compared to findings in other vertebrates and relevance to disorders affecting color processing is discussed.

## Zebrafish Retinal Structure and Development

Zebrafish retina, like those of other vertebrates, contains five neural types organized into layers. Distal-most are photoreceptors (PRs) with cell bodies in the outer nuclear layer (ONL) and terminals forming synaptic contacts with the dendritic processes of bipolar (BCs) and horizontal (HCs) cells in the outer plexiform layer (OPL). The cell bodies of these second order neurons, as well as those of third order amacrine cells (ACs), reside in the inner nuclear layer (INL). BCs are presynaptic to AC and ganglion cell (GC) dendrites in the inner plexiform layer (IPL), where processes in the distal IPL, or sublamina *a*, mediate OFF-type responses and processes in sublamina *b* mediate ON-type responses. GC bodies are in the most proximal layer, the GC layer (GCL).

### Development of Neurons and Circuits

Eye morphogenesis in zebrafish begins at 12 h postfertilization (hpf; Schmitt and Dowling, 1994). Subsequent structural changes to eye primordia result in well-formed optic cups at 24 hpf (Schmitt and Dowling, 1994), and development of the neural retina progresses in direction from the inner (vitreal) to the outer (scleral) retina. GC and AC form first (~32 hpf), with a small number of GC axons leaving the eye to form the optic nerve at ~34–36 hpf (Stuermer, 1988; Schmitt and Dowling, 1994, 1999; Burrill and Easter, 1995). Initial differentiation of PRs occurs in a patch ventral to the optic nerve head at ~50 hpf (Kljavin, 1987; Raymond et al., 1995; Hu and Easter, 1999; Schmitt and Dowling, 1999; Raymond and Barthel, 2004). Development in the patch continues in advance of other retinal regions until ~70 hpf, when the entire retina appears homogeneous. BC are the last cell type to form at ~60 hpf (Schmitt and Dowling, 1999) and between 60–70 hpf all neuronal cell types can be identified and synapses are apparent (Schmitt and Dowling, 1999). The vertical pathway (i.e., PR-to-BC-to-GC) appears functional at 70–74 hpf (Schmitt and Dowling, 1999), corresponding to innervation of the optic tectum (Stuermer, 1988), hatching, and the onset of visually guided behaviors (Easter and Nicola, 1996, 1997). Many of the developmental transcription factors such as cone rod homeobox gene (*crx*), atonal (*ath5*) and thyroxin β2 nuclear receptor (*trβ2*), which are key to retinal development in mammals, also operate in zebrafish retina (Kay et al., 2001; Shen and Raymond, 2004; Jusuf et al., 2011; Suzuki et al., 2013).

### Development of Electrical Signals

Physiological studies show cone inputs dominate electroretinogram (ERG) responses in zebrafish larvae younger than days postfertilization (15 dpf; Bilotta et al., 2001) and recordings from larval GC already reveal complex cone-opponent spectral responses (Connaughton and Nelson, 2015). Rod PR density is low in larvae younger than 10 dpf (Fadool, 2003), explaining the cone-dominated electrical activity of the larval eye. Increased rod outer segment length and synaptic connections occur between 12 dpf and 15 dpf (Branchek and Bremiller, 1984) resulting in an increase in sensitivity (Branchek, 1984). However, the retina does not appear adult-like until after 20 dpf (Branchek and Bremiller, 1984) at which time both rod and cone responses can be detected in the zebrafish ERG (Bilotta et al., 2001). The early development of cones, and later development of rods, is also characteristic of mammalian retinas (Carter-Dawson and Lavail, 1979).

## Cone Photoreceptors Allow Detection of Light Ranging from Long (Red) to Ultraviolet (UV) Wavelengths

### Opsins and Cone Morphology

Zebrafish possess four morphological cone types as well as rods. The cones cover an even broader range of the optical spectrum than do human cones. In adults, the cones are classified as short single cones, long single cones, and a double cone pair based on morphology (Engström, 1960), opsin expression, and relative peak absorption wavelengths (λmax). Short single cones have a λmax ~360 nm; these are the ultraviolet (UV)-sensitive cones (SWS1 opsin). Long single cones are short wavelength sensitive (SWS2 or blue opsin) cones with a λmax ~415 nm. Double cones include an accessory member, the middle wavelength sensitive (MWS or green) cone, with a λmax ~480 nm (probably green opsin RH2-2), and a principal member, the long wavelength sensitive (LWS1 or adult red opsin) cone, with a λmax ~570 nm (Nawrocki, 1985; Robinson et al., 1993; Cameron, 2002; Chinen et al., 2003; Allison et al., 2004; Endeman et al., 2013). Molecular analysis of opsin expression in these different cone types has identified two genes encoding the opsins in red cones, four genes for green opsins, and a single gene each for the blue and UV opsins (Chinen et al., 2003).

Within each PR outer segment, a chromophore (11-*cis*-retinal) binds to an opsin molecule forming the visual pigment complex that absorbs light photons (Hubbard and Kropf, 1958). There are two forms of retinal, derived from either vitamin A1 or A2 (Saari, 2012). Zebrafish use only vitamin A1 based pigments, unless they are experimentally treated with thyroxine, which induces the synthesis of vitamin A2 (Allison et al., 2004; Enright et al., 2015).

### Cone Mosaics in Larvae and Adults

**Figure 1:**
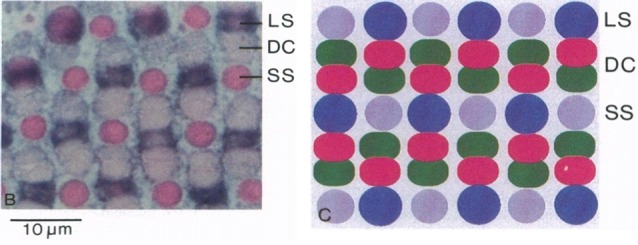
Zebrafish cone mosaic. In adult zebrafish, the cone mosaic is highly structured, with red and green double cones (DC) between blue (Long single, LS) and UV (Short single, SS) cones, with the red cone always next to the blue cone and the green cone always next to the UV cone (from Robinson et al., 1993, National Academy of Sciences, U.S.A. Reprinted with permission).

The four cone types allow zebrafish to respond to wavelengths of light ranging from UV to red. In adults (Figure 1), the cones are organized into a row mosaic (Allison et al., 2010) in which double cones alternate with single cones such that the red cone is always adjacent to a blue cone and the green cone is always adjacent to a UV cone (Engström, 1960; Larison and Bremiller, 1990; Raymond et al., 1993; Allison et al., 2010). This row mosaic, first described by Engström (1960), supports a two Red: two Green: one Blue: one UV cone ratio that is constant over the entire retina, except for a small primordial region (Allison et al., 2010). Rod PRs insert into this cone mosaic, with four rods forming a square around each UV cone (Fadool, 2003; Morris and Fadool, 2005). When the retina is viewed in cross-section, it is evident that rods and cones do not form a single row but organize into tiers within the PR layer. The nuclei of short single UV cones are located most proximally. Moving distally one next identifies nuclei of long single blue cones, and then the double cones. Rod nuclei are located proximal to short single cones, while their outer segments are distal to double cones (Branchek and Bremiller, 1984; Robinson et al., 1993).

In contrast to adults, the cone mosaic in larval zebrafish (Larison and Bremiller, 1990) is less well organized and heterotypic, with regularly spaced PRs, but no row pattern (Allison et al., 2010). UV cones form first (Raymond et al., 1995; Robinson et al., 1995; Schmitt and Dowling, 1999) and are most abundant in larval retina, followed by blue cones. Red/green double cones form last (Robinson et al., 1995; Schmitt and Dowling, 1999) and are least abundant (Allison et al., 2010). Interestingly, opsin expression displays the opposite pattern with expression of red and blue opsins occurring several hours before expression of UV opsin (Schmitt and Dowling, 1999). Red and blue opsins occur in a tandem genetic pattern under a single promoter (Chinen et al., 2003), as do the four variants of green opsins. In cone types with multiple opsin genes (i.e., red and green cones), *in situ* hybridization identified sequential expression of these opsin genes during development (Takechi and Kawamura, 2005). It is the shorter-wavelength-peaking LWS2 that is mainly expressed in larvae, while adults express a mixture of LWS1 and LWS2. For green opsins, the shorter-wavelength-peaking RH2-1 is earliest expressed, followed by the longer-wavelength-peaking RH2-2, RH2-3 and RH2-4. After the cone mosaic is present, the rod mosaic forms (Fadool, 2003; Morris and Fadool, 2005). Transition from larval to the adult mosaic occurs during the postlarval/juvenile period when the fish are >3 weeks of age (Allison et al., 2010).

## Outer Retina—Processing of Color Signals at the First Retinal Synapse

Processing of color signals from different PRs occurs in outer retina. BCs are morphologically diverse retinal interneurons that receive combinations of inputs from rods and different cone types. Zebrafish HCs similarly contact specific combinations of PR types resulting in mono- and multiphasic spectral response properties that in total reflect inputs from all four cone types. PR inputs to BCs are modified by feedforward and feedback synapses from these spectrally-coded HCs, resulting in significant color processing at this first retinal synapse.

### Vertebrate HCs

Mammalian retinas have, in general, two morphological HC types. A-cells are axonless, and large in dendritic extent; whereas the dendritic fields of B-cells are smaller and an axon ending in a terminal arbor projects from the cell body (Fisher and Boycott, 1974). Primate and rodent retinas are exceptions to the mammalian pattern. In primates, HI HCs are similar to B-cells, HII HCs are similar to A-cells, but HIII HCs more resemble teleost HCs, with a long “axon” without terminal arborization projecting from the cell body (Ahnelt and Kolb, 1994; Dacey et al., 1996, 2000). In rodents, there is only a single, B-type HC morphology (Peichl and González-Soreiano, 1994). In teleosts, all HC types bear an axon lacking terminal arborization. There are four HC morphologies in teleosts: H1–H4. Types H1–H3 are postsynaptic to cone PRs, while H4, the rod horizontal cell (HC), is postsynaptic only to rods (Stell and Lightfoot, 1975; Stell, 1975; Weiler, 1978).

In vertebrates, each HC type receives input from specific numbers and types of PRs resulting in either spectrally monophasic (L-type) or multiphasic (C-type responses; Nelson, 1977; Yang et al., 1983; Siminoff, 1986; Djamgoz et al., 1988; Negishi et al., 1988; Dacey et al., 1996, 2000; Asi and Perlman, 1998; Twig and Perlman, 2004; Yin et al., 2006). One conspicuous difference in the physiology of mammalian HCs as compared to other vertebrates is the complete lack of C-type responses.

### Cone Contacts of Horizontal Cell Types

Zebrafish HC contribute to color vision by facilitating color-opponent mechanisms through feedback and feedforward connections to PR and/or BC (Song et al., 2008). As in goldfish (Stell, 1967; Stell and Lightfoot, 1975), zebrafish cone-contacting HCs are morphologically characterized as H1, H2, or H3 types, and rod horizontal cells (RHCs or H4 type) which contact rods (Song et al., 2008; Li et al., 2009). All zebrafish HC are axon-bearing, with a snake-like terminal that does not contact PRs running within the OPL (Song et al., 2008).

**Figure 2:**
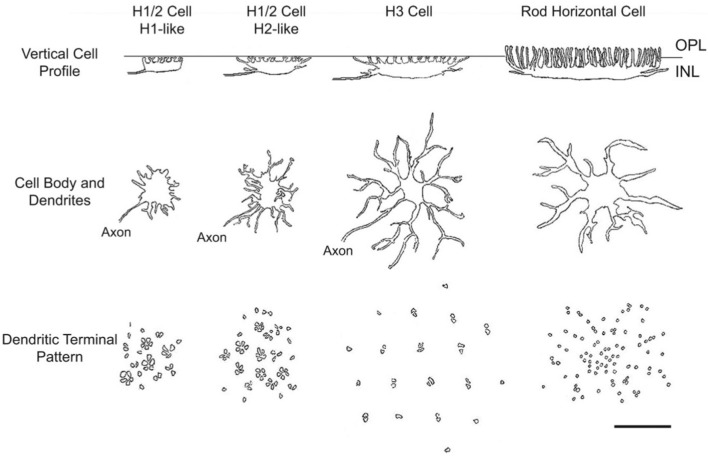
Zebrafish horizontal cells (HCs). HCs in zebrafish retina are morphologically distinct. H1-like cells are smaller in vertical profile and somal size compared to H2 and H3 cells, and all types have distinct dendritic connections with presynaptic photoreceptors (PRs). Reproduced with permission from Song et al. (2008).

Zebrafish H1 HC (Figure 2) have a plate-shaped soma from which short dendritic processes protrude. H2 HC exhibit a wider profile than H1 HC and have an ellipsoid soma that extends tendril-like dendritic processes. The dendritic terminals of H1 and H2 occur in clusters of 5–6 boutons arranged in a rosette. The rosettes contact both double and single cone rows. When overlapped onto this cone mosaic, patterns consistent with contacts between HCs and either red, green and blue cones (H1 cells) or green, blue and UV cones (H2 cells) appear (Li et al., 2009). Due to H1/2 cell partial clusters and in some cases single boutons, it is probable certain individual cones may make connections with more than one HC. The remaining elements of the cluster would be contacted by another HC. H3 HC have an elongated cell body, with 3–4 long dendritic processes terminating in a rhomboid pattern with boutons arranged in doublets (Connaughton et al., 2004; Song et al., 2008). The dendritic extent of H3 is distinctly wider than either H1 or H2, making it easily recognizable. Examination of the H3 dendritic pattern on a transgenic cone mosaic where UV cone terminals are labeled suggests H3 cells selectively contact long single blue and/or short single UV cones, a pattern appearing to serve short wavelengths (Song et al., 2008; Li et al., 2009).

### Development of H3 Types

The development of H3 cells was followed in larval zebrafish where the ratio of UV to blue cone synapses increased from about 2:1 at 3 dpf to 5:1 at 10 dpf. This ratio is greater than the ratio of UV to blue cones, suggesting H3 cells actively seek out contacts with UV cones during development. The *tr*β2 nuclear receptor is required for red cone development (Ng et al., 2001; Suzuki et al., 2013). When the density of UV cones was increased (and red cones decreased) by suppression of *tr*β2 nuclear receptor, H3 dendrites grew and accommodated the extra UV cells with synapses. Genetic suppression of blue or UV synaptic transmission suggested that, at least for the UV to H3 synapse, activity is critical for synapse formation (Yoshimatsu et al., 2014).

### Horizontal Cell Spectral Processing

**Figure 3:**
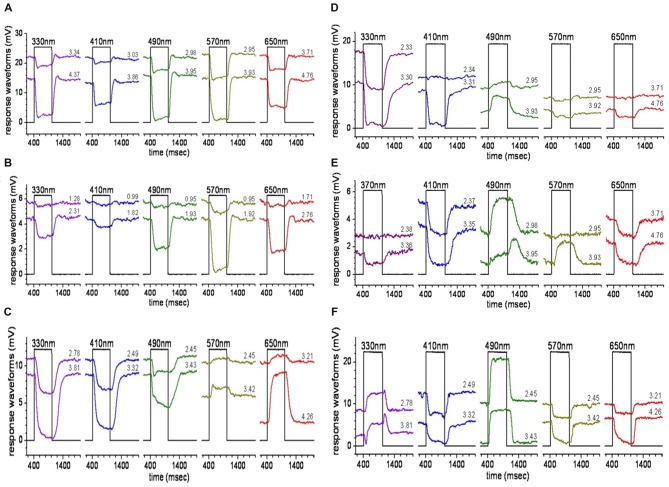
HC responses. Stimulation of zebrafish eyecups with light of different spectral wavelengths evokes different responses from zebrafish HCs. **(A,B)** H1-type cells hyperpolarize to all wavelengths, giving an L-type response; while **(C)** H2 cells are biphasic, hyperpolarizing to G, B, and ultraviolet (UV) and depolarizing to R light. H3-type HCs, in contrast, display triphasic **(D,E)** and tetraphasic **(F)** responses to spectral stimuli (from Connaughton and Nelson, 2010).

Intracellular recordings of HC spectral responses identified luminosity (L-type) and chromaticity (C-type) HCs (Connaughton and Nelson, 2010), as in other teleosts (Svaetichin and Macnichol, 1958), including other cyprinidae (Naka and Rushton, 1966a, b; Kaneko, 1970). L-type, or spectrally monophasic responses hyperpolarize to light at all stimulus wavelengths and irradiances (Figures 3A,B). Most zebrafish HCs encountered (~66%) were L-type. Of these L-type responses, the majority were red-preferring L2 cells, with peak sensitivity to wavelengths ~560 nm; the rest are red/green L1 cells with a mean peak sensitivity ~490 nm (Connaughton and Nelson, 2010). C-type responses included spectrally biphasic, triphasic, and tetraphasic varieties. Biphasic cells depolarized to long wavelength stimulation (>570 nm) but hyperpolarized to wavelengths ≤530 nm (Figure 3C). Spectral responses of triphasic cells, in general, hyperpolarized to long wavelength stimuli (650, 610, and 570 nm), and depolarized to middle and short wavelengths (530, 490, and 450 nm). UV triphasic cells hyperpolarized strongly to 370 and 330 nm stimuli, while blue triphasic cells hyperpolarized weakly to these UV stimuli, but more strongly to a 410 nm stimulus (Figures 3D,E). The final HC spectral response identified was a unique tetraphasic response (Figure 3F) characterized by hyperpolarization to red-yellow (650–570 nm), depolarization to green and green-blue (530, 490 nm), hyperpolarization to 450, 410 and/or 370 nm, and depolarization to UV (330 nm; Connaughton and Nelson, 2010). Dye fills of recorded cells indicate HCs with L-type and biphasic spectral responses were similar in morphology (Connaughton and Nelson, 2010) and distinct from cells with triphasic and tetraphasic responses. Combining images of microelectrode stains and response properties with the anatomical studies of HC morphologies and PR terminals (Li et al., 2009), suggests: (a) L-type responses occur in H1 HCs; (b) biphasic cells are H2 cells; and (c) triphasic and tetraphasic responses occur in H3-type HC (Li et al., 2009; Connaughton and Nelson, 2010).

## Cone Synapses With Bipolar Cells

### Vertebrate BCs Are Similar in Glutamatergic Mechanisms and Axonal Stratification Patterns

Zebrafish BCs were initially classified by their glutamate-gated currents, which identified both ON- and OFF-type cells. ON-cells express metabotropic glutamate receptors and a chloride channel forming glutamate transporter on their dendritic arbor (Grant and Dowling, 1995); whereas, OFF-type cells express AMPA/kainate receptors (Connaughton and Nelson, 2000). The ON-BC dendritic “glutamate transporter channel” is not seen in mammals as it provides presynaptic inhibition on axon terminals of mammalian ON-type BCs (Wersinger et al., 2006). Morphologically, the axon terminals of physiologically ON-BCs were found within IPL sublamina *b* while OFF-BC terminals were in sublamina *a* (Connaughton and Nelson, 2000). These sublamina divide the IPL roughly in half, with sublamina *a* represented by the outer half, near ACs, and sublamina *b* by the inner half, near GCs, consistent with IPL structure and BC morphology in other vertebrates. Subsequent studies using dye-labeling methods (DiI) introduced a 6-stratum scheme for classifying bipolar axon terminal depth within the zebrafish IPL and identified ~17 morphological types of BCs based on axonal stratification patterns (Connaughton et al., 2004; Li et al., 2012). This number of types was large as compared to mammals (Kolb et al., 1981; Haverkamp et al., 2005; Masland, 2012) and amphibians (Wu et al., 2000), but similar to the 15 types identified in goldfish with Golgi staining (Sherry and Yazulla, 1993). Of the 17 types in zebrafish, ~7 were OFF-cells, ~6 were ON-cells, and ~4 were presumed ON-OFF cells with axon terminals in both sublamina *a* and *b* (Connaughton et al., 2004). This latter type, with bistratified axon terminals, physiologically expressed either OFF-type or ON-type glutamate receptors (Connaughton and Nelson, 2000).

### Bipolar Cells Differentially Contact Multiple Cone Types

**Figure 4:**
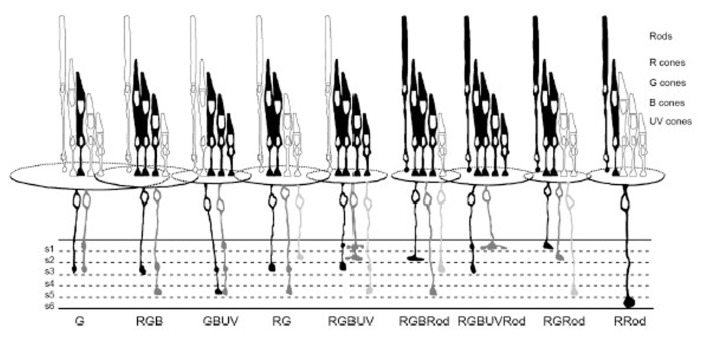
Bipolar cells make diverse connections with PRs. Summary figure (reproduced with permission from Li et al., 2012) showing the different PR connections associated with morphologically-distinct types of BCs. Each BC was identified by axon terminal ramification within the six sublaminae (s1–s6) of the inner plexiform layer (IPL) and by dendritic connections with specific cone types. Most BC types make connections with multiple (>2) PR types, except for two types that exclusively contact green cones (at left of figure) Rod, contacts rods; R, red cones; G, green cones; B, blue cones; UV, UV cones. Shading reflects frequency of identification of a given type, with the darker (black) color indicating the most commonly observed type.

Superposition of DiI-stained BC dendrites onto the zebrafish cone mosaic revealed BC type-specific synaptic connection patterns with PRs, and further expanded the number of BC types to ~33 (Li et al., 2012). Nine dominant patterns of PR connections accounted for 96% of a total of 18 patterns found (Figure 4). These dominant patterns included both cone-only BCs, exclusively postsynaptic to cone PRs, and mixed-rod-cone input BCs (Li et al., 2012). Only one of the cone-selective patterns was restricted to a single cone type (green cones), and one of the mixed rod-cone patterns received input from all PR types. Examining the axon-terminal stratification types among these PR connectivity patterns, six of the nine patterns included monostratified ON types, with terminals in s4–s6 and either monostratified or bistratified OFF types, with all terminals in s1–s3. Four of the patterns included ON-OFF axon terminal stratification types. The variety in presynaptic connections, the dendritic field size of each BC, and the terminal ramification patterns in the IPL (Li et al., 2012) suggest selective and elaborate color circuits involving BCs across the entire retina.

### Bipolar Cell Spectral Properties

**Figure 5:**
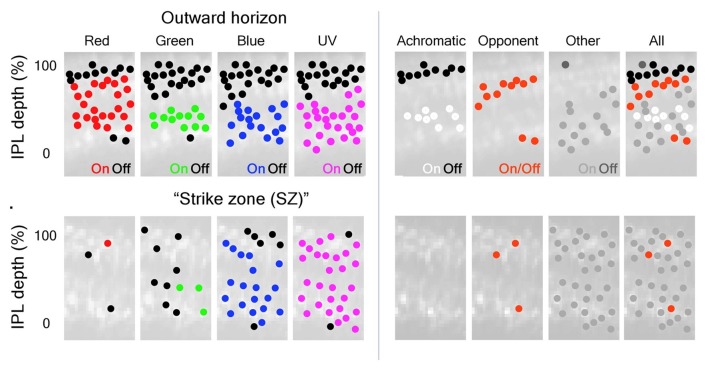
Color inputs to inner retina are segregated in the IPL. BC terminals in the larval zebrafish IPL can be segregated based on the spectral inputs they respond to across the visual scene. (Left) BC-terminal excitation by R, G, B, and UV stimuli are represented as red, green, blue and magenta dots located throughout the IPL; inhibitory responses are represented as black dots. The distribution of ON-responses varies based on the spectral stimulus and which part of the visual scene (outer horizon vs. “strike zone (SZ)”) is being viewed. The “SZ” represents looking forward and upward at prey items. (Right) Subsequent classification of the different spectral inputs from BCs into the IPL identified achromatic and color-opponent regions in this synaptic layer that were most evident outside the “SZ”. For the right panels: black dots = achromatic OFF-responses, white dots = achromatic ON-responses, red dots = color-opponent responses, gray dots = “other” (taken from Figure 3, Zimmermann et al., 2018).

Recent calcium-imaging 2-photon microscopy of BC axon terminals in live zebrafish larvae found BCs that were chromatic (wavelength selective), achromatic (responding to all wavelengths), or opponent (opposite responses at two wavelengths; Figure 5). To image light-driven calcium activity, a transgene, consisting of the ribeyeA/ctbp2 promoter, which is selectively expressed by retinal BCs, and the synaptophysin-GCaMP6f fusion protein, a synaptically localized calcium sensor (Dreosti et al., 2009; Zimmermann et al., 2018), was inserted into the zebrafish genome.

A blue-UV chromatic response was found to be regionally selective, with a preponderance of ON-type UV-excited BC terminals throughout the IPL. In retinal topology, these cells densely occupy a temporal-ventral “strike zone (SZ),” the region looking forward and upward at prey (Zimmermann et al., 2018). Both ON and OFF achromatic types responded with excitation or inhibition to all wavelengths tested. Opponent responses included red excited, green, blue and UV inhibited, as well as blue and UV excited, but red, green inhibited. These results are broadly consistent with neuroanatomical connectivity of BCs, particularly in respect to the many cone signals represented in BC terminals throughout the depth of the IPL, which attests to the idea that multi-cone contacting BCs may connect to cones in a color-opponent manner.

To date, there have been no studies reporting the spectral responses of zebrafish BCs using either whole-cell patch or sharp electrode techniques. In the closely related Giant Danio retina (*Danio aequipinnatus*), however, patch recordings of bistratified cone BCs with axonal boutons in both sublamina *a* and sublamina *b* (Cab BCs) revealed color-opponent responses, excited by one wavelength, but inhibited by another. The Cab cells subtract signals from cones with different opsin expression, and multiple spectral patterns were observed. There was evidence that different types of BC dendritic glutamate receptors were stimulated by different spectral types of cone (Wong and Dowling, 2005). Further, spectral light stimulation (red, green, or blue), evoked double color-opponent responses. Selectively blocking AMPA/kainate receptors or glutamate transporter associated chloride channels on these cells revealed that the responses to stimulation with short and long wavelengths are mediated by these two-different glutamate-gated mechanisms (Wong and Dowling, 2005).

### ERG b-Waves Suggest Bipolar-Cell Spectral Properties

The ERG is a light-evoked retinal field potential that sums activities of retinal neurons. ERG b- and d-waves represent, and are dominated by, the massed activity of ON- and OFF-BC types in zebrafish, respectively. The b-wave in adult zebrafish receives contributions from all four cone types and exhibits evidence of red-green color-opponent mechanisms (Hughes et al., 1998). Modeling these inputs, (Cameron, 2002) separated b-wave signals into four different processing channels, one for each cone type. Two color channels (long and middle wavelengths) were involved in color-opponent processing, while the short and UV wavelength channels were not (Cameron, 2002). This suggests BCs detect wavelength-dependent differences in both chromatic and luminance contrast (Cameron, 2002).

ERG b-wave responses in larval zebrafish are different from those in adults. Adult ERG responses include a-wave, b-wave, and d-wave components at all stimulus wavelengths and irradiance levels (Bilotta et al., 2005). The components present in a larval ERG, however, are wavelength dependent. For example, in response to UV light stimulation, the larval ERG includes a large a-wave, delayed b-wave, and small/no d-wave. However, if middle/long wavelength stimuli are used, the ERG has a small/no a-wave followed by large amplitude b- and d-waves (Bilotta et al., 2005). The ERG of larval zebrafish retina is dominated by UV cones, and the physiological differences in a-wave amplitude evoked using UV vs. longer wavelength stimuli can be attributed to differences in cone representation in larval responses (Bilotta et al., 2005). However, differences in larval b-wave (ON-BC) responses may also be due to differences in glutamate receptor expression on these cells. For example, UV and short wavelength stimuli seemed to be mediated by APB-sensitive metabotropic glutamate receptors on ON-BC dendrites, while longer wavelength stimuli were not (Bilotta et al., 2005). Multiple glutamate-gated mechanisms are present on adult zebrafish ON-BCs (Connaughton and Nelson, 2000; Nelson and Singla, 2009) and it appears that the mechanisms may be somewhat selective for cone type.

## Spectral Processing in Amacrine Cells

### AC Characteristics

As a group, and across all vertebrates, ACs are the most diverse class of retinal neuron. ACs are categorized by their physiological response to light, their great variety of neurotransmitter expression as determined using immunolabeling, their dendritic morphology, and dendritic lamination patterns in the IPL. The different types of ACs range from 43 in roach (Wagner and Wagner, 1988) to 22 in cat (Kolb et al., 1981) to ~30 in rabbit (MacNeil et al., 1999; Masland, 2012). Physiological responses of ACs are similarly diverse. Light stimulation evokes sustained or transient, ON, OFF, or ON-OFF responses in both mammalian (Nelson, 1982; Nelson and Kolb, 1985; Stafford and Dacey, 1997; Menger and Wässle, 2000) and non-mammalian (Kaneko, 1970, 1973; Pang et al., 2002; Miller et al., 2006; Zhang and Wu, 2010) retinas.

Structural and functional diversity is also present in zebrafish ACs where ~28 morphological types have been identified in 5 dpf larval retina (Jusuf and Harris, 2009). Many larval AC types are also present in adults, as determined by dye (Connaughton et al., 2004) and/or immunolabeling (Marc and Cameron, 2001; Yazulla and Studholme, 2001; Arenzana et al., 2006; Yeo et al., 2009; Jang et al., 2011). These cells exhibit narrow, medium, or wide field dendritic arbors (Jusuf and Harris, 2009; Lewis et al., 2015) and are widely distributed over the entire retina (Yeo et al., 2009; Torvund et al., 2017); although it appears the density of parvalbumin s4, and tyrosine hydroxylase (dopaminergic) types is greater in temporal-ventral, or ventral retina than nasal retina (Yeo et al., 2009; Jang et al., 2011). In adult zebrafish, AC light responses are either transient or sustained, with excitation observed at light ON, light OFF, or both light ON and light OFF (Torvund et al., 2017). Most dendritic branching patterns are highly planar and narrowly restricted to single strata within the IPL. Both monostratified types (one branching plane) and bistratified types (two branching planes) are observed (Connaughton et al., 2004). Diffuse dendritic branching through multiple IPL strata was uncommon.

### ON-OFF Amacrine Cells Select Red Cone Signals

**Figure 6:**
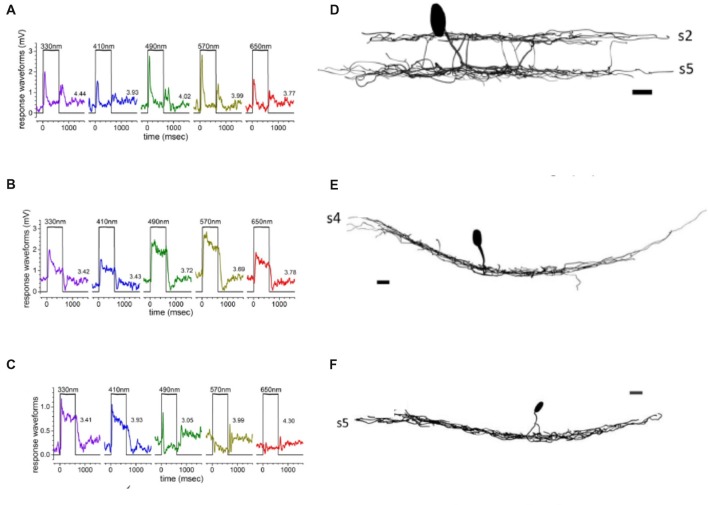
Spectral responses of ACs are associated with specific cell types. Transient ON-OFF amacrine cells (AC) responses **(A)** were characteristic of bi-stratified ACs with dendrites in both the OFF- and ON-sublaminae **(B)**. In contrast, sustained ON responses **(C)** were observed in ACs with a dendritic arbor monostratified in sublamina *b* **(D)**. Spectrally multiphasic C-type responses were primarily observed in ON-cells, as shown in the biphasic response **(E)** from a monostratified AC with dendrites in s5 of sublamina *b* **(F)** (Torvund et al., 2017).

ON-OFF responses in zebrafish are characteristic of all bistratified ACs (Figures 6A,B). Most of these have processes in both IPL sublamina *a* and *b*, though some ON-OFF cells have processes bistratified only within sublamina *a* (Torvund et al., 2017). These cells respond to spectral stimuli ranging from 330 nm to 650 nm; however, modeling of their responses indicates virtually exclusive input from red cones (Torvund et al., 2017), both at light ON and light OFF. For calcium imaging, ACs can be marked by the inhibitory-interneuron selective transgene *ptf1a:gal4* (Jusuf and Harris, 2009). Marked cells express the synaptic calcium reporter transgene *UAS:SyGCAMP3* (Rosa et al., 2016). In the IPL, bands of ON-OFF responding processes and synapses can be found in both sublamina *a* and sublamina *b*, a result consistent with the existence of ON-OFF bistratified AC types. This method, however, is not able to directly link both bands to actions from single bistratified ACs. The frequency responses of these ON-OFF signals included different populations peaking at either ~5 Hz or ~10 Hz.

### ON-Sustained and OFF Amacrines Sum Red and Green Cone Signals

Zebrafish OFF-type ACs, like ON-OFF types, receive dominant input from red cones, with lesser input from green or blue cones (Torvund et al., 2017). These cells are monostratified in either s1 or s2 of sublamina *a*. ON-type AC, with monostratified dendrites in s4, or rarely s3, receive mixed luminosity signals from red and green cones, leading to mid-spectral sensitivity peaks (Figures 6C,D). The ON ACs were sustained types, not excited at light OFF, and contributions from blue and UV cones are noticeably lacking in these non-opponent responses (Torvund et al., 2017). The dendrites of both ON and OFF *ptf1a* ACs, marked for synaptic calcium responses, labeled distinct IPL strata, with a major ON band in sublamina *b*, and a major OFF band in sublamina *a*. In addition, calcium imaging suggests a lower intensity representation of both these response types throughout retinal depth (Rosa et al., 2016). Crossover GABAergic inhibition from ON-ACs appears to shape the frequency response characteristics of OFF-BC terminals (Rosa et al., 2016), leading to the speculation that sustained ON-ACs contribute “low pass” characteristics to OFF-BC boutons.

### C-Type Amacrine Cells Subtract Cone Signals

Unique as compared to mammals, some zebrafish ACs are color-opponent. UV cone inputs contribute significantly to color-opponent (C-type) responses in zebrafish AC. The dendrites of all these C-type ACs ramify deep in sublamina *b*, indicating they are ON-type cells morphologically, though physiologically the response sign depends on wavelength (Figures 6E,F). In adult retina, both spectrally biphasic and triphasic AC responses are present, as also observed in HC spectral responses (Connaughton and Nelson, 2010). Biphasic AC responses include blue-yellow and red-green units. Red-green opponent cells depolarized to red, but hyperpolarized to green, blue and UV stimulation; while blue-yellow opponent cells responded with a sustained depolarization to short wavelength stimulation, but hyperpolarized to long wavelengths (Torvund et al., 2017). Some triphasic ACs depolarized to UV, hyperpolarized to blue, and depolarized to green. Interestingly, inclusion of (marginally significant) red cone inputs to the triphasic response would cause it to be reclassified as a tetrachromatic AC response (Torvund et al., 2017). Calcium imaging studies have found color-opponent BC terminals within the layer of dendritic branching of color-opponent ACs (Zimmermann et al., 2018). The bistratified, color-opponent Cab bipolar of Giant Danio also sends a terminal bouton to this same layer (Wong et al., 2005). Taken together these studies identify a potential synaptic input for C-type ACs. Wide dendritic fields, sometimes seen with dye coupling, suggest a wavelength-dependent modulatory role.

## Morphology and Physiology of Retinal Ganglion Cells

### GC Physiology in Vertebrates

As the output neuron of the retina, there have been many studies documenting light responses of retinal GCs. In fact, color-opponent GCs have been documented in a variety of species, including, but not limited to, monkey (De Monasterio and Gouras, 1975; Zrenner et al., 1983; Dacey and Lee, 1994; Calkins et al., 1998; Sun et al., 2006; Crook et al., 2009; Lee and Sun, 2009; Dacey et al., 2014; Silveira et al., 2014), cat (Daw and Pearlman, 1970; Crocker et al., 1980; Guenther and Zrenner, 1993), wallaby (Hemmi et al., 2002), rabbit (Caldwell and Daw, 1978; De Monasterio, 1978; Mills et al., 2014), guinea pig (Yin et al., 2009), mouse (Chang et al., 2013), turtle (Bowling, 1980; Rocha et al., 2008), chick (Zhou et al., 2005), and fish (Wagner et al., 1960; Witkovsky, 1965; Daw, 1968; Raynauld, 1972; Spekreijse et al., 1972; Van Dijk and Spekreijse, 1984; Mackintosh et al., 1987; Bilotta and Abramov, 1989; Sakai et al., 1997). Therefore, the presence of spectrally selective GC responses in zebrafish would not be surprising. A wide range of GC characteristics/response types makes sense as these cells receive, integrate and process information from BC and AC about various parameters of the visual scene.

### Zebrafish GC Morphology, Development and Tectal Projection

**Figure 7:**
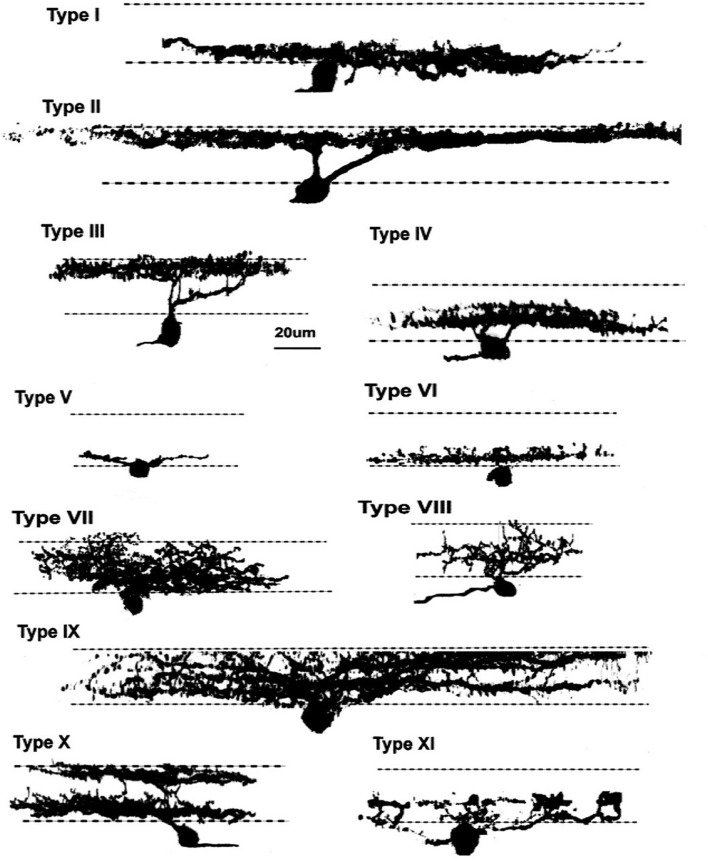
Zebrafish ganglion cells (GCs). Di-I labeling of GCs in whole mount tissue identified 11 morphological types, based on dendritic arborization patterns in the IPL. These patterns included processes restricted to 1–2 sublaminae, as well as more diffuse arborization patterns.Reproduced with permission from Mangrum et al. (2002).

Mammals, in general, have a greater number of morphological types of GCs (Kolb et al., 1981; Sanes and Masland, 2015) than zebrafish (Mangrum et al., 2002). The 11 types identified in adult zebrafish (Figure 7) are grouped as either wide-field (2), narrow-field (4), multistratified (3), or diffuse (2) based on dendritic extent and patterns of stratification (Mangrum et al., 2002). A main reason for less types in zebrafish is a closer grouping of cell body diameters (5–8 μm) and dendritic field diameters (80–200 μm) as compared to mammals (cat: 10–40 μm cell bodies; 20–900 μm dendritic fields; (Kolb et al., 1981). A wide spread of metrics allows for more type-features to be distinguished. In mouse retina, similar to zebrafish, GC dendritic fields are restricted in range (150–450 μm; Sun et al., 2002). The greatest density of zebrafish GCs occurs in a temporal-ventral patch similar to the “SZ”, a visual space used for feeding (Mangrum et al., 2002; Zimmermann et al., 2018).

Time-lapse imaging experiments in larval zebrafish (Mumm et al., 2006) discovered diverse dendritic growth patterns and laminar targeting mechanisms used by GCs in the IPL, resulting in 15 stratification patterns. GC input to the tectum is somewhat segregated, with inputs relaying similar information (such as stimulus direction) going to similar tectal areas (Johnston and Lagnado, 2012). Though these inputs to the tectum are functional by 66 hpf, it is not until 78 hpf that tectal neurons begin to display mature responses. This suggests that GCs are capable of information processing before structural development in the retina is complete (Niell and Smith, 2005).

### Ganglion Cell Light Responses

**Figure 8:**
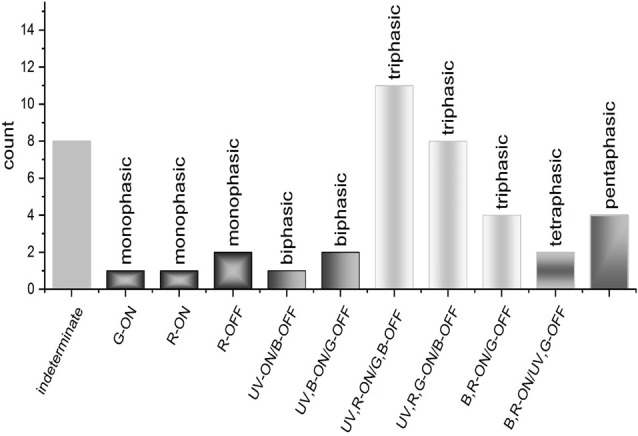
GC spectral responses. Loose patch recordings from larval zebrafish GCs revealed a diversity of response types, most of which are spectrally multiphasic. Triphasic responses were the most abundant type identified and could be subdivided into three distinct spectral patterns. Bi-, tetra-, and pentaphasic responses were also recorded (Connaughton and Nelson, 2015).

Light responses of larval zebrafish GCs are either transient or sustained, with ON-, OFF- and ON-OFF subtypes (Emran et al., 2007). Most ON-OFF cells are bistratified in sublaminae *a* and *b*, while ON- and OFF-cells are monostratified (Zhang et al., 2010). Between 2 dpf and 4 dpf zebrafish GCs undergo depolarizing to hyperpolarizing shift in GABAergic inhibitory E_Cl_ (Zhang et al., 2010), a process common to vertebrate early neuronal development (Li et al., 1998). Stable light responses are recorded as early as 4 dpf (Zhang et al., 2010) and color-opponent and non-opponent responses are evident at 5 dpf and 6 dpf, when responses are dominated by spectrally multiphasic types (Connaughton and Nelson, 2015). The most common spectral type is the triphasic GC response with bursts of spikes to both long and short wavelength stimuli, but inhibited firing at middle wavelengths. Other multiphasic responses include biphasic, tetraphasic and pentaphasic units (Connaughton and Nelson, 2015). Like adult ACs, red cone inputs are prominent in larval GC spectral responses. However, most GC responses include significant and commonly dominant, UV excitation, resulting in multiphasic spectral properties. Very few larval zebrafish GCs are spectrally monophasic (Figure 8). In adult zebrafish, light-evoked spike discharges are seen in the optic nerve, particularly ON-OFF types (Li and Dowling, 2000; Huang et al., 2005), but the spectral properties are not known.

## Wavelength Processing Circuitry in Zebrafish

The different subtypes of second- and third-order neurons in zebrafish receive inputs from single or, more often, multiple cone PRs, resulting in spectrally monophasic and multiphasic responses. As a result, the signals from each cone type are relayed, through both feedforward and feedback circuitry, throughout the entire retina. One feature of this intermixing of cone signals is both synergistic and antagonistic interactions between different cone signals in distal neurons, such as HC and BC, a feature also noted in AC and GC of the inner retina. The spectral properties of GCs, retinal output neurons, are similar to the color-opponent spectral responses of HCs, suggesting one of the origins of GC spectral processing lies in distal retina, with the cone connectivity patterns of HC and BC. One expects there is further selective integration of BC and AC physiological types onto the dendrites of GC receptive fields in the IPL, resulting in even greater diversification and refinement of spectral properties. The dominance of red cone signals in spectrally monophasic AC types is reminiscent of the L-type signals from H1 HCs, suggesting the existence of an L-type BC pathway. Despite the evidence for a mixing of cone signals in the image-processing circuits of the inner retina, existing evidence suggests that signals from the 4 retinal cone types in zebrafish are not treated equally.

### Red Cone Signals

Red cones are only 1/3 of adult cone types. Nonetheless, red cone signals dominate spectral responses in HC and AC. These red (LWS) signals are relayed directly from the principal member of the double cone to H1 HCs(Li et al., 2009) resulting in red-dominated monophasic (L1- or L2-type) physiologies in 2/3 of HCs studied (Connaughton and Nelson, 2010). Even though contacting them, H1 HCs do not receive physiological signals from blue (SWS2) cones, and do not contact UV (SWS1) cones at all. LWS cones are included in seven of nine of BC connectivity patterns or 87% of 321 BCs studied (Li et al., 2012), potentially accounting for the prevalence of red cone signals seen in ACs (Torvund et al., 2017). However, these BC pathways, while contacting red cones, are not red cone selective, as green (MWS) cones overlap, comingling in all but one of these same BC connectivity patterns and 93% of BCs studied (Li et al., 2012).

Non-color-opponent AC responses are either selective for or dominated by red cone signals; these cells are OFF-stratified, ON-stratified, and ON-OFF bistratified cells, altogether accounting for 89% of recorded cells (Torvund et al., 2017). The variety of AC stratification patterns in this group suggest that AC processes receiving LWS-selective cone inputs are present throughout the depth of the IPL (Torvund et al., 2017), consistent with direct input from red cone contacting, and physiologically red cone dominated BCs, also presumably of multiple stratification types. In contrast to adult ACs, LWS inputs to GCs in larval zebrafish retinas contribute mainly to spectrally multiphasic responses. For the latter, red cone inputs are always excitatory (Connaughton and Nelson, 2015), but the responses are dominated by excitation from UV cones. The contrast between dominant AC and GC red cone spectral patterns presents a dilemma, and it is unclear at present how large a role larval development plays, as b-wave spectral sensitivity progresses from UV-dominated in larvae to a more broadly sensitive pattern in juveniles and adults (Saszik et al., 1999). A reasonable conjecture is that both non-opponent and opponent BC pathways exist for red cone signals. This idea is consistent with the finding of a population of red cone opponent ACs in adults with a unique stratification pattern (Torvund et al., 2017).

### Green Cone Signals

Despite a numerical representation in adults identical to red cones, green cone signals are not dominant in zebrafish physiology. In the outer retina, while red cones dominate H1 HC responses, UV cones dominate one physiological subtype of H3, and blue cones dominate the other, there is no HC strictly devoted to excitation from green cones. These signals appear as complementary components in H1 HC, particularly the L2-type response and H2 HC biphasic signals, where they combine with blue cone signals. The most selective and prominent role of green cone signals in the outer retina are as the inhibitory (depolarizing) elements in triphasic and tetraphasic HC responses (Connaughton and Nelson, 2010).

In ACs, green cone inputs to non-color-opponent cells were small and found mainly in sustained-ON AC, with processes monostratified either in the OFF sublamina or in the distal-most layer of the ON sublamina (Torvund et al., 2017). Green cone signals were seldom identified in the numerically dominant ON-OFF types. No ACs that selectively represent green cone signals have been encountered (Torvund et al., 2017). The relative absence of MWS signals in ACs is mysterious, unless green cone-to-BC synapses are commonly either inactive, inhibitory, or opponent. In calcium imaging physiology of BC boutons (Zimmermann et al., 2018) it does appear that the most common form of BC color opponency is red excitatory, green inhibitory (Figure 5). These red-ON, green-OFF boutons localize mainly in s2, s3 of IPL sublamina *a*. Green cones are the only type that selectively innervate BCs, in fact there are three varieties of OFF stratifying BCs that only contact green cones (Li et al., 2012). The green cone signals appear to be largely inhibitory (Connaughton and Nelson, 2015), and speculatively, the inhibition might involve OFF-type, or color-opponent OFF-type, green cone BCs. However, in adults, the spectrally triphasic, green cone inhibited, color-opponent AC did not co-stratify with green cone BC terminals in sublamina *a*.

### Blue and UV Cone Signals

In outer retina, blue and UV cone signals are represented in about 1/3 of recorded HC. Blue (SWS2) cones contact H1, H2 and H3 HC (Li et al., 2009), but only contribute signals to biphasic (H2) and blue-preferring triphasic (H3) responses (Connaughton and Nelson, 2010). UV cones directly connect only to H3 cells (Li et al., 2009). However, UV inputs to H3 cells appear variable, as this morphological type can generate three different spectral responses (Connaughton and Nelson, 2010): (1) UV-excited spectrally triphasic responses dominated by UV cone excitation; (2) tetraphasic (UV-inhibitory); and (3) blue-preferring triphasic responses excited by both UV and blue cones. Spectrally multiphasic patterns are presumed evidence of feedback to blue and UV cones from HC types receiving stimulation from LWS cones (Li et al., 2009; Connaughton and Nelson, 2010) to generate HC multiphasic responses.

In adult inner retina, stimulation of blue and UV cones contributes to biphasic and triphasic responses in zebrafish ACs, a pattern similar to outer retina. These include a UV-depolarizing blue-yellow opponent biphasic unit and UV-depolarizing triphasic responses (Torvund et al., 2017). Short wavelength inputs to larval GCs elicit spectrally triphasic responses, similar to triphasic HC responses (Connaughton and Nelson, 2015). Interestingly, adult ACs with C-type responses have processes exclusively in sublamina *b*, suggesting a functional segregation of short wavelength BC-to-AC synapses in the IPL. Adult connectomic analysis identified 3 BC types directly postsynaptic to UV cones with axon terminals in sublamina *b*, and 5 BC types postsynaptic to blue cones (Li et al., 2012), suggesting there are a relatively few, possibly selective, circuits that relay short wavelength signals to inner retina, and that short wavelength cone signals are commonly conveyed in a color-opponent pathway.

### Stratification of IPL Cone Signals

There may be some segregation of cone signals in the zebrafish IPL. BC connectomics provides pathways for red, green, blue and UV cone signals into all IPL strata. This homogeneity is partly enforced by BCs with multistratified axon terminals, as well as the prevalence of BCs contacting multiple cone types (Li et al., 2012). The finding of dominant red cone signals in all non-opponent ACs, regardless of stratification pattern, is in accord with a distribution of L-type, or achromatic, red cone signals throughout the IPL (Torvund et al., 2017). Nonetheless, there are some imbalances. The RRod type BC conducts red cone (and rod) signals only, and projects to IPL sublamina *b*. The solely green cone contacting BCs project only to s1 and s3 of sublamina *a*, with most boutons in s3 (Li et al., 2012). Depolarizing sustained ACs stratify in s3-s4. In addition to red cone signals, these cells are strongly excited by green cone stimulation (Torvund et al., 2017). Over 60% of BC types project to sublamina *a* (Li et al., 2012), a curious imbalance. In calcium imaging studies of BC boutons, Zimmermann et al. (2018) found that the IPL strata containing achromatic OFF-BC boutons may be much narrower than previously supposed, localized to s1 and not all strata of sublamina *a*. The remaining s2-s3 band contains color-opponent BC boutons of various types. There is also an s5 color-opponent band, perhaps innervating the color-opponent ACs that branch in s5 (Torvund et al., 2017). Torvund et al. (2017) proposed that the multistratified GBUV BC types (Li et al., 2012) might be the color-opponent pathway innervating color-opponent ACs, a theory yet to be tested but consistent with the color-opponent BC axon terminal distribution (Wong and Dowling, 2005; Zimmermann et al., 2018). Stratum s4 and thereabouts is the true achromatic ON-type band. Zimmermann et al. (2018) find that IPL banding varies with retinal region as well, with UV excited boutons being broadly prevalent in the “SZ”. The indications are that there are multiple functional layers in zebrafish IPL, both for chromatic and achromatic processing.

## Comparison With Mammalian Color Circuitry

In mammals, there are well-defined, specific inner and outer retinal circuits for rod and cone signals. Rod signals are relayed through rod BCs (RBCs) to AII ACs and then to ON-GCs through gap junctions with ON-BC terminals (Kolb and Famiglietti, 1974). Cone signals are relayed through cone BCs to GCs (Kolb, 1970). As true for most fish, zebrafish have mixed input (mb) BCs, a close analog of mammalian RBCs, but receiving inputs from both rods and cones. In mammals, cones synapse on HC dendrites, and rods synapse on HC axon terminals (Nelson et al., 1975; Kolb, 1977; Dacheux and Raviola, 1982). In zebrafish, rods synapse on a separate HC type, while cones selectively innervate 3 distinct HC types and 4 different BC types (Li et al., 2012). Overall, it appears that rod and cone signals in zebrafish, like mammals, are selectively sampled by retinal interneurons. Zebrafish HCs are either spectrally monophasic (red cone signals) or multiphasic (color opponency among cone signals, (Connaughton and Nelson, 2010); while only spectrally monophasic patterns appear in mammalian HCs. Mammalian HC axon terminals are physiologically rod selective, while the mammalian HC cell bodies mix red cone, rod and UV/blue cone signals (Steinberg, 1969; Dacheux and Raviola, 1982; Nelson, 1985; Dacey et al., 1996).

There are BCs selective for UV cones in mammals (Mariani, 1984; Haverkamp et al., 2005). In contrast, there is no anatomically UV cone selective BC in zebrafish (Li et al., 2012). Nonetheless, UV-selective BC physiology is likely present (Saszik et al., 2002; Zimmermann et al., 2018). Zebrafish GC responses include short-wavelength color-opponent responses, as reported in other species. In primate retina, for example, the blue-ON/yellow-OFF response of small bistratified GCs has been particularly well described (Dacey, 1999; Marshak and Mills, 2014). This spectrally multiphasic response is due to feedback circuitry involving HCs in outer retina. In inner retina, it is attributed to separate inputs from ON- and OFF-BCs, which themselves are postsynaptic to different cone types, as well as chromatic AC inputs (Marshak and Mills, 2014). It is likely that the multiphasic responses dominated by UV signals observed in larval zebrafish GCs similarly integrate and reflect inputs from UV-selective outer retinal circuitry involving HCs and direct inputs from spectrally selective presynaptic BCs and/or ACs.

## Color Preference, Perception and Behavior

Zebrafish display color discrimination or preference when given a choice between different sections of a tank that have different background colors (Colwill et al., 2005; Risner et al., 2006; Avdesh et al., 2012; Ahmad and Richardson, 2013; Oliveira et al., 2015; Park et al., 2016). Most studies report a natural preference for blue/short wavelength light in both adult (Colwill et al., 2005; Risner et al., 2006; Bault et al., 2015; Oliveira et al., 2015; Peeters et al., 2016) and larval (Park et al., 2016; Peeters et al., 2016) fish. A few studies, in contrast, report a natural preference for red/long wavelength light (Avdesh et al., 2012; Ahmad and Richardson, 2013), which may be associated with food/foraging and be related to color of prey items (Spence and Smith, 2008).

In behavioral testing, color preference is best explained by a multiple-mechanisms model that includes both non-opponent (UV, B) and opponent (G-B and G-R) systems (Risner et al., 2006). This model is also most accurate in describing spectral sensitivities of ERG b-wave and ON-tectal responses in zebrafish (McDowell et al., 2004), suggesting behavioral responses reflect physiological processing of the visual scene. Interestingly, though color choice experiments indicate zebrafish can behaviorally distinguish color stimuli, the optomotor response (OMR), a standard vision-based behavior used to identify mutant strains, is “color blind” (Krauss and Neumeyer, 2003; Orger and Baier, 2005) as it is dominated by LWS (red) cones in adults (Krauss and Neumeyer, 2003) and pooled inputs from red and green cones in larvae (Orger and Baier, 2005). OMRs can be reduced, though, when UV or B cones are selectively ablated (Hagerman et al., 2016), suggesting short wavelength cones do contribute to the OMR, possibly in an inhibitory manner (Krauss and Neumeyer, 2003; Hagerman et al., 2016).

## Zebrafish as Models of Retinal Disease

There have been several recent reviews detailing the applicability of zebrafish to the study of ocular/retinal diseases (Gross and Perkins, 2008; Bibliowicz et al., 2011; Link and Collery, 2015). These reviews highlight the experimental strengths of zebrafish: similar retinal/eye anatomy with humans, rapid development and large clutch size, easy manipulation and phenotype observation, and amenability of genome editing techniques to generate mutant and transgenic lines (Bibliowicz et al., 2011; Link and Collery, 2015). Of the >20 diseases for which zebrafish are used, ~50% target PRs/PR layer (Link and Collery, 2015), and 13 specific genes associated with PR degeneration have been identified (Brockerhoff and Fadool, 2011). Behavioral studies using the optokinetic response (OKR; Brockerhoff et al., 1995), the escape response (Li and Dowling, 1997), or the visual motor response (Emran et al., 2008), coupled to ERG recordings allowed functional assessment of mutant phenotypes. Interestingly, many of the mutations leading to PR degeneration are not associated with transduction, but with protein trafficking (Brockerhoff and Fadool, 2011), consistent with the high metabolic demand and almost-constant functioning of retinal PRs. Here, we focus on those zebrafish lines with mutations in cone PRs. These studies have largely been performed with larval (5–7 dpf) zebrafish because, at this age, the cone-rich retina contains few/no rods (Holzhausen et al., 2009).

### Mutations Affecting All Cone Types

The OKR is a visually-guided behavior based on saccadic eye movements as a stationary animal tracks a moving grating. This is a reliable test that can be elicited as early as ~4 dpf in zebrafish (Brockerhoff et al., 1995; Neuhauss, 2003). Brockerhoff et al. (1995, 1997, 1998) were the first to apply this technique to the identification of mutant phenotypes. Most of these behaviorally-identified zebrafish were later found to have mutations in cone-specific genes.

One of the first mutants identified was *no optokinetic response a (*noa*)*. As the name suggests, *noa* larvae do not display an OKR. These mutants have ERG recordings with normal a-waves, but abnormal b-waves (Brockerhoff et al., 1995), consistent with altered glutamate responses in postsynaptic BCs (Connaughton, 2001). Subsequent, molecular analysis identified two alleles of *noa: m631* and *a13* (Taylor et al., 2004). *noa*^a13^ mutants (originally *no optokinetic response b*, (Taylor et al., 2004) have a deficient *dihydrolipoamide-S-acetyltransferase* (*dlat*) gene, resulting in the absence of the E2 subunit of the enzyme pyruvate dehydrogenase (PDH; Taylor et al., 2004). PDH is a critical enzyme involved in ATP production in mitochondria because it catalyzes the reaction that converts pyruvate to Acetyl Co A (Karp, 1999). Absence of this enzyme results in PDH deficiency, an inherited metabolic disorder (Barnerias et al., 2010; Patel et al., 2012). The *dlat* (*noa*^a13^) mutant is, therefore, a model for human PDH deficiency.

*no optokinetic response c (nrc)* mutants, in addition to an absent OKR, have a diverse phenotype that includes floating ribbons in cone pedicles (Allwardt et al., 2001) and reduced numbers of unevenly distributed synaptic vesicles (Van Epps et al., 2004). Abnormal ERG recordings (Allwardt et al., 2001) revealed a light adaptation defect in distal retina (Van Epps et al., 2001). ON-GC responses in inner retina are also reduced/absent (Emran et al., 2007). Molecular analysis identified the *nrc* mutation as a premature stop codon in the *synaptojanin 1 (SynJ1)* gene (Van Epps et al., 2004) in cone PRs (Holzhausen et al., 2009). *Synaptojanin-1* is strongly expressed in brain^1^ and the synaptojanin-1 protein is involved in endocytosis of clathrin-coated vesicles (Perera et al., 2006) in nerve terminals.

A cone-specific mutation is also observed in zebrafish *no optokinetic response f* (*nof*) mutants. *nof* fish have a nonsense mutation in the gene coding for the α-subunit of cone transducin (TαC; Brockerhoff et al., 2003). Development of the transgenic line *TG*(3.2Tα*CP-EGFP*) allowed visualization of TαC in all zebrafish cone types, making *nof* mutants a model for achromatopsia (Kennedy et al., 2007). Interestingly, though *nof* cones lack transducin, they do respond to bright light stimuli, albeit at a much-reduced level (Brockerhoff et al., 2003).

A mutation in the cone cGMP-phosphodiesterase α subunit (*pde6α^1^* or *pde6c*), also causes cone degeneration (Stearns et al., 2007; Nishiwaki et al., 2008) in zebrafish. cGMP-PDE is an enzyme complex located in PR outer segments that is part of the phototransduction cascade (Rodieck, 1998). In mouse rods, absence/mutations in either the α (Huang et al., 1995), β (Pittler and Baehr, 1991), or γ (Tsang et al., 1996) subunits of cGMP-PDE result in rod degeneration. cGMP-PDE in zebrafish cones is similarly required as mutations lead to cone-specific degeneration.

#### Mutations/Treatments Affecting Specific Cone Types

The generation of knockout (KO) and mutant lines has also identified genes specific to a given cone type. The transcription factor *Tbx2b*, for example, is necessary for UV cone formation (Alvarez-Delfin et al., 2009) as a mutation in *tbx2b* results in the conversion of UV cones to rods, resulting in the *lots-of-rods (lor)* phenotype (Alvarez-Delfin et al., 2009). Though *lor* zebrafish have a strong OKR, UV opsin expression in larval retinas is deficient. All other cone types are present and *tbx2b* mutants have an even and abundant distribution of rods over the entire retina. Similarly, the transcription factor *sine oculis homeobox homolog 7* (*six7*) is required for green cone development (Ogawa et al., 2015). PRs in *six7* KO larvae don’t express any of the four, green opsin (RH2-1 to RH2-4) genes, resulting in a loss of green cones that persists to adulthood. Interestingly, the *six7* KO phenotype also includes reduced blue opsin expression (larval stage), increased number of rods (larvae), and a switch in red opsin expression (adults; Ogawa et al., 2015). Finally, red cone fate is determined by thyroid hormone receptor β2 (*trβ2)* expression (Suzuki et al., 2013) as knockdown of *trβ2* results in a loss of red cones, but an increase in UV cones (Suzuki et al., 2013). Red cone loss is also selective in *partial optokinetic response b (*pob*)* mutants (Brockerhoff et al., 1997). *pob* zebrafish are red color blind due to rapid degeneration of red cones. PR loss in *pob* is due to a mutation in the *pob* gene, which codes for a protein required for red cone survival (Taylor et al., 2005).

Allison and colleagues have developed transgenic zebrafish lines in which a single class of PR can be chemically ablated (Fraser et al., 2013; Hagerman et al., 2016). These lines are key in determining how removal of one cone type may alter the existing PR mosaic as well as how the retina will respond. The zebrafish retina continues to grow throughout the life of the fish and is able to regenerate cells in response to damage (reviewed in Brockerhoff and Fadool, 2011). Chemical ablation of a single cone type in adult retina can trigger retinal regeneration (Fraser et al., 2013; Hagerman et al., 2016). For example, selective ablation of UV cones stimulates the formation of new UV cones, at a higher density than predicted (Fraser et al., 2013). This recovery and regeneration is rapid: behavioral responses (OMR) are restored in 72 h (Hagerman et al., 2016). Selective loss of blue cones, however, results in an even faster regenerative response, with behavioral responses restored in 24 h (Hagerman et al., 2016). The rapid regeneration capabilities of zebrafish retinal tissue, and the ability of this tissue to respond to the selective loss of a given cone type, is an important step in determining mechanisms that could be used to restore cell loss after retinal degeneration.

Finally, other studies have induced diseases with retinal complications using non-molecular mechanisms. For example, prolonged hyperglycemia, characteristic of diabetes, can be induced by either immersing zebrafish in a glucose solution (Gleeson et al., 2007; Alvarez et al., 2010) or through streptozotocin injection (Olsen et al., 2010; Intine et al., 2013), both of which result in changes to the retina, making zebrafish a model for diabetic retinopathy. In addition to retinal thinning (Gleeson et al., 2007; Olsen et al., 2010) and changes to retinal vasculature (Alvarez et al., 2010), there are also marked changes to cone PRs in hyperglycemic zebrafish retinas with double cones displaying the most severe alterations (Alvarez et al., 2010).

## Conclusion

Zebrafish are capable of rich color processing due to the presence of four cone types that allow detection of light ranging from UV to red. After stimulation of the phototransduction cascade in cones, spectral signals are immediately processed and modified in outer retina through synapses with HC. BC signaling relays spectral information to inner retina, where multiple feedforward inputs converge on AC and GC and feedback inputs from AC further modify spectral signals. These ON-signals are faithfully relayed to the tectum. As a result, zebrafish are able to behaviorally discriminate color and display innate color preferences. This elaborate signaling, coupled to well-known retinal anatomy and targeted genetic manipulation, has resulted in zebrafish that exhibit mutation-induced red blindness (Brockerhoff et al., 1997), UV blindness (Alvarez-Delfin et al., 2009), and green blindness (Ogawa et al., 2015). Zebrafish also serve as models for vision disorders, such as diabetic retinopathy (Gleeson et al., 2007; Alvarez et al., 2010), retinitis pigmentosa (Baye et al., 2011), achromatopsia (Kennedy et al., 2007) and Leber’s congenital amaurosis (Baye et al., 2011), making zebrafish a model of choice for study of human retinal disorders.

## Author Contributions

AM, RN and VC all contributed to writing the article and selection of figures. Supported by funds from the College of Arts and Science and the American University Library (to VPC).

## Conflict of Interest Statement

The authors declare that the research was conducted in the absence of any commercial or financial relationships that could be construed as a potential conflict of interest.

## Footnotes

^1^https://www.ncbi.nlm.nih.gov/gene/8867
